# Extended reality as a means to enhance public health education

**DOI:** 10.3389/fpubh.2022.1040018

**Published:** 2022-11-23

**Authors:** Ying-Chiang Jeffrey Lee, Bryce Puesta Takenaka

**Affiliations:** ^1^Department of Molecular Biology, Princeton University, Princeton, NJ, United States; ^2^Department of Social and Behavioral Sciences, Yale School of Public Health, New Haven, CT, United States

**Keywords:** public health, education, technology, augmented reality, virtual reality, extended reality (XR), training

## Abstract

Technology has transformed the classroom and learning environments. From electronic whiteboards to tablet computers, educators now have access to a multitude of tools that enhance the learning experience. Educational technologies that rely on extended reality (XR) such as augmented and virtual reality are being used, or suggested for use, in various settings and often focus on technical fields such as medicine, dentistry, and aviation. Here, we propose that XR can be used in public health education to better prepare both undergraduate and graduate trainees for real world, complex public health scenarios that require public engagement, investigative skills, and critical decision making. Several opportunities for XR use are outlined that provide perspective on how XR can supplement traditional classroom instruction methods by providing an immersive, participatory training environment. XR offers an opportunity for public health students to gain confidence, have repeated simulated exposures in a safe and equitable environment, and build competency in critical functions they will likely perform as future public health professionals.

## Introduction and current uses of XR in education

There is a need for public health education reform in the United States and across the globe. Complex public health challenges have arisen ranging from infectious disease outbreaks and biothreats, such as SARS-CoV-2/COVID-19 to gun violence and ambiguity surrounding public health guidance. For the next generation of public health professionals to meet these challenges, they will need reforms in educational approaches that include the use of new educational technologies (1). Augmented reality (AR) and virtual reality (VR) are two arms of extended reality (XR) approaches that are being explored as educational training tools (2). AR can be deployed in several formats, including from smartphones and tablets using onboard cameras to the more immersive wearables such as the Microsoft HoloLens 2. AR builds upon the existing physical reality surrounding the user and projects images and text onto that environment. The user retains full situational awareness of their surroundings. On the other hand, with VR, the user's entire vision is encompassed by a headset, creating an alternate, virtual, space that the user interacts with. Several companies are currently in, or planning on entering, the VR market including Oculus, Sony, and Apple. The third component of XR, includes mixed reality that interfaces between AR, VR, and the real world, but for simplicity and comprehensiveness, the term XR will be used in the remainder of this article to encompass all these technologies.

With the additional technological advancements and use of digital and mobile platforms across the public sector, XR has sparked interest due to its potential contributions to the space of education across all levels and has been found create a more immersive and effective learning experience for students (3–5). For example, classrooms have been able to experience 3D spaces to document museum artifacts and ancient heritages to better understand the past (6). XR has also been associated with improved learning motivation, interest, creativity, academic performance as well as being a time-efficient alternative to traditional methods of instruction and learning (7–11).

XR technology is being implemented as training tools in medical training, military live fire weapons applications, as well as sports education (12–17). Most of these uses of XR technology are for technical skills that require extensive use of psychomotor faculties, but a broader approach to XR in education reform can include its use in fields and situations that require a mixture of soft and hard skills—public health is one such field. Public health is a unique discipline that blends aspects of medicine with the social sciences, yet while physicians and other health professionals require hours of skills training through residency programs and social workers require hours of in-person experience, no such extensive training exists for public health students. Educational reform is needed to better prepare public health students and trainees for encountering real world situations. XR presents as a potentially disruptive technological tool that can be used in public health education.

## Opportunities for XR use in public health education

XR integration into public health education can enhance learning by providing students with opportunities to access environments, situations, and tools that are beyond the reach of traditional didactic instruction (18). Previous use of XR in medical education have resulted in higher academic achievement compared to conventional teaching methods (19). These positive results may translate into public health education. XR would bridge the gap between theory taught in the classroom and practical experience gained in the real world. A course could integrate XR into its standard curriculum or, designed into dedicated Skills Labs that emphasize participatory approaches using XR and emphasize the repeated use of XR for a set of particular scenarios. Such courses could include lectures followed by intensive hands-on training using XR in an interactive and iterative format. Here, we provide three different example cases of training simulations and role-playing scenarios that public health professionals may encounter depending on their career path. While these are case examples, each public health program should tailor XR use to their own individual needs, curriculum material, and focus areas.

### Case #1—Outbreak investigation

The initial stages of an outbreak investigation can include tracking patients, searching through medical records, interviewing exposed and control individuals, and identifying causal pathways (20). While this may be taught in specific career roles and job functions after the student has left academia and entered the workforce, academic instruction on outbreak investigation, and the best practices associated with such work, can provide students with early exposure and develop an understanding of what thorough investigative work comprises in a safe environment.

XR can provide an immersive experience for students and help build competency in identifying key aspects of a public health investigation and has been suggested for use in infectious disease emergency management (21). Simulations of past outbreaks on an XR platform can allow students to consider what actions are appropriate for a given situation or circumstance that is presented at a point in time. Multiple options for actions programmed to set outcomes along decision paths could result in the student gaining a deeper understanding of the outbreak, identifying infectious pathways, and performing contact tracing at the population level to not only identify key spreaders but also perhaps identify possible origins of an outbreak. Student actions could also result in the mishandling of the investigation and resulting in the outbreak spreading uncontrollably. The benefit of using simulations built upon, or even just closely following, past events is that we understand what approaches worked and what did not with the advantage of hindsight. Students will gain experience in making smarter investigative choices and filter out extraneous noise, in the form of excess information that would slow down an investigation. On the other hand, XR simulations could also be programmed for hypothetical outbreak investigations that combine features and details from a number of different outbreaks and even include unexpected variables that have not been seen before. This approach could help students deal with entirely novel situations, modulate stress, and become more mentally agile. These hypothetical and imaginary outbreak investigations can also serve as learning platforms where professionals can examine what decisions are made by trainees, why such decisions were made, and what the possible outcomes could be. These results could then feed into developing educational material, even plotting out human behavior and decision making during public health investigations.

### Case #2—Red teaming and simulations of crisis response

Red teaming approaches have been widely used in the United States for issues regarding national security, including biological risks, to probe for vulnerabilities and forecasting threats (22). These events are often performed as tabletop exercises and include actors playing key individuals and stakeholders that respond to a certain biothreat to model what could happen in a real-world event. While red teaming is often largely confined to indoor spaces, it could also be conducted in a real-world setting in a natural environment, similar to how training is conducted for certain mass casualty incidents by healthcare providers and first responders. A limiting factor here is the extent of participation, especially by students, and resources needed to enact a certain event in the real-world.

XR would allow students in red team events to “experience” the health threat they are analyzing and even allow for a digital record keeping and diagramming of group decision making as they move through the red team exercise. XR technology will allow for red team student participants to be situated in different locations. Events that are written into the red team exercise storyline could also be digitally displayed to provide a more realistic context. This could help create interactive representations of the issue being analyzed through the red team exercise. By providing visual context, public health trainees could respond in a more realistic manner and better highlight the vulnerabilities that exist. Students using XR would also be able to access the exercises with more freedom and with more repetitions than a real-world training event would practically allow. Far less resources would also be required, potentially saving on capital and materials that would be allocated for a multi-day real world training event that incorporates first responders and healthcare providers.

### Case #3—Public engagement by public health leadership

Public health professionals occupying various levels of health leadership will need to communicate effectively, often to a common audience, and be able to assure, reinforce, and distribute accurate public health messages to diverse audiences. These public speaking situations could present in the form of a press conference, television interview, or briefing groups of first responders or staff at various levels of government. Current public health education curricula do not include skills training on public speaking and such a program would greatly increase the speaking competency of public health professionals. A pertinent example is the messaging around vaccines during the COVID-19 pandemic and communicating with those who are vaccine hesitant. Trainees who have had experience in these situations will enter the workforce with a better sense of how to prepare and act.

While a traditional classroom role playing exercise could mimic such public engagements, XR can bring a completely new dimension to this form of training. Using XR, students could be presented with a room full of reporters or members of the community and filled with the lights and sounds typically found in a presentation setting. Such sensory stimuli will provide a realism that would be difficult to replicate in a classroom. Avatars in the XR space or even real students in the room can ask questions and engage in dialogue with the student trainee. Instructors would be able to provide feedback on how the student performed and offer areas of improvement before the student is immersed in the XR simulation again.

## Conclusion

Public health education can be enhanced through the innovative use of educational technologies, specifically applying XR for developing critical competencies and skills needed in public health roles. The examples we have included in this article are meant to demonstrate the diverse applications of skillet training that XR can offer. From simulating disease outbreak investigations to red teaming crisis response and public engagement, XR greatly expands the toolkit available to educators to train the next generation of public health professionals. Despite the promises of XR, there are several limitations to overcome before XR can be widely adopted in public health education, and education in general. Some limitations previous studies have found in XR, specifically AR, use include implementation issues, the lack of teacher training, few educational experiences, lack of conceptual foundation, limited educational research, and lack of institutional support in addition to present optical challenges (23, 24).

Barriers to the adoption of XR in general also include issues around its economics, detail capture, and initial curriculum adaptation. From an economic and purchasing perspective, initial costs for public health programs to purchase or even rent XR headsets could be a major barrier. Academic institutions are hesitant on the cost-effectiveness of investing in these tools (25). Associated costs would not only include maintenance of the devices but also the educational material fees unless the programs have their own XR design and programming team. While costs are a potential limitation to XR use, the very nature of public health work could itself play a role in undermining the effectiveness of XR as a public health educational tool. Many of the competency domains identified by the Public Health Foundation require a combination of technical and soft skills. To include these competencies in Skills Labs with XR during simulations will require extensive development and could be a hurdle to designing effective XR experiences. Lastly, if issues of cost and XR experience development are resolved, public health students will have to contend with hardships around integrating XR into standard public health Skills Lab courses. Studies have documented challenges such as obtaining tool licensing, procuring headsets and other hardware for XR, and the technical literacy to operate these specialized software (26). In addition, systematic reviews of XR implementation have also identified the time-consuming development phase as well as teacher resistance and reduction in imagination as challenges toward using XR in education (27). Furthermore, it is important to acknowledge the national divide in the digital technology that continues to disproportionately exclude Black and Brown learners (28, 29). Providing XR within the classroom may be an opportunity to shorten the access and uptake to technology gap. Institutions that integrate XR should also be mindful to ensure instructors and faculty are able to appropriately transition these tools into their classroom (30).

Despite these challenges, XR is a disruptive technology that has the potential to fundamentally transform public health education. Further studies into how XR can be designed and deployed for maximum reach and effectiveness can benefit the spread and acceptance of XR in curricula. XR provides an immersive and simulated approach toward training students on hands on, participatory techniques and can lead to generations of highly competent public health professionals.

## Data availability statement

The original contributions presented in the study are included in the article/supplementary material, further inquiries can be directed to the corresponding author.

## Author contributions

YCL conceptualized the manuscript topic. YCL and BT contributed to researching, writing, and editing of this work. All authors contributed to the article and approved the submitted version.

## Funding

This publication was supported by the Princeton University Library Open Access Fund.

## Conflict of interest

The authors declare that the research was conducted in the absence of any commercial or financial relationships that could be construed as a potential conflict of interest.

## Publisher's note

All claims expressed in this article are solely those of the authors and do not necessarily represent those of their affiliated organizations, or those of the publisher, the editors and the reviewers. Any product that may be evaluated in this article, or claim that may be made by its manufacturer, is not guaranteed or endorsed by the publisher.
